# 'Tis Easy to Die

**Published:** 1882-01

**Authors:** 


### "'TIS EASY TO DIE."

IF I had strength to hold a pen, I would write how easy and delightful it is to die, were the last words of the celebrated surgeon, Wm. Hunter; and Louis XIV. is recorded as saying, with his last breath, "I thought dying had been more difficult."

That the painlessness of death is owing to some benumbing influence acting on the sensory nerves may be inferred from the fact that untoward external surroundings rarely trouble the dying.

On the day that Lord Collingwood breathed his last the Mediterranean was tumultuous; those elements which had been the scene of his past glories rose and fell in swelling undulations, and seemed as if rocking him to sleep. Captain Thomas ventured to ask if he was disturbed by the tossing of the ship. "No, Thomas," he answered, "I am now in a state that nothing can disturb me more —I am dying, and I am sure it must be consolatory to you and all that love me to see how comfortably I am coming to my end." In the *Quarterly Review* there is related an instance of a criminal who escaped death from hanging by the breaking of the rope. Henry VII. of France sent his physician to examine him, who reported that after a moment's suffering the man saw an appearance like fire, across which appeared a most beautiful avenue of trees. When a pardon was mentioned the prisoner coolly replied that it was not worth asking for. Those who have been near death from drowning, and afterward restored to consciousness, assert that the dying suffer but little pain.

Captain Marryatt states that his sensations at one time when nearly drowned were rather pleasant than otherwise. "The first struggle for life once over, the water closing around me assumed the appearance of waving green fields. It is not a feeling of pain, but seems like sinking down, overpowered by sleep, in the long, soft grass of the soft meadow."

Now, this is precisely the condition presented in death from disease. Insensibility comes on, the mind loses consciousness of external objects, and death rapidly and placidly ensues from asphyxia.

